# Development and Validation of a Simple Convenience Store SHELF Audit

**DOI:** 10.3390/ijerph15122676

**Published:** 2018-11-28

**Authors:** Tanya M. Horacek, Elif Dede Yildirim, Erin Kelly, Adrienne A. White, Karla P. Shelnutt, Kristin Riggsbee, Melissa D. Olfert, Jesse Stabile Morrell, Anne E. Mathews, Terezie T. Mosby, Tandalayo Kidd, Kendra Kattelmann, Geoffrey Greene, Lisa Franzen-Castle, Sarah Colby, Carol Byrd-Bredbenner, Onikia Brown

**Affiliations:** 1 Department of Public Health Food Studies and Nutrition, Syracuse University, Syracuse, NY 13244, USA; ekkelly@utica.edu; 2 Human Development and Family Studies, Auburn University, Auburn, AL 36849, USA; elifdy@auburn.edu; 3 Biology Department, Utica College, 1600 Burrstone Rd., Utica, NY, 13502, USA; 4 School of Food and Agriculture, University of Maine, Orono, ME 04469-5735, USA; awhite@maine.edu; 5 Department of Family, Youth and Community Sciences, University of Florida, Gainesville, FL 32611, USA; kpagan@ufl.edu; 6 Department of Nutrition, University of Tennessee, Knoxville, TN 37996, USA; scolby1@utk.edu (S.C.); kolmstea@vols.utk.edu (K.R.); 7 Division of Animal & Nutritional Sciences, School of Agriculture, West Virginia University, Morgantown, WV 26506, USA; Melissa.Olfert@mail.wvu.edu; 8 Department of Molecular, Cellular & Biomedical Sciences, University of New Hampshire, Durham, NH 03824, USA; jesse.morrell@unh.edu; 9 Food Science and Human Nutrition Department, University of Florida, Gainesville, FL 32611, USA; anne.mathews@ufl.edu; 10 Department of Food Science, Nutrition, and Health Promotion, Mississippi State University, Starkville, MS 39762, USA; terezie.mosby@msstate.edu; 11 Department of Food, Nutrition, Dietetics and Health, Kansas State University, Manhattan, KS 66506, USA; martan@k-state.edu; 12 Health and Nutritional Sciences Department, South Dakota State University, Brookings, SD 57007, USA; kendra.kattelmann@sdstate.edu; 13 Department of Nutrition and Food Sciences, University of Rhode Island, Kingston, RI 02881, USA; ggreene@uri.edu; 14 Department of Nutrition and Health Sciences, University of Nebraska-Lincoln, Lincoln, NE 68583, USA; lfranzen2@unl.edu; 15 Department of Nutritional Sciences, Rutgers University, New Brunswick, NJ 08901, USA; bredbenner@sebs.rutgers.edu; 16 Department of Nutrition, Dietetics & Hospitality Management, Auburn University, Auburn, AL 36849, USA; onb0001@auburn.edu

**Keywords:** environmental audit, fruit vegetable assessment, consumer food environment

## Abstract

*Background* This paper describes the development, reliability, and convergent validity of a practical tool—the Convenience Store Supportive Healthy Environment for Life-Promoting Food (SHELF) Audit. *Methods* Audit items included: a variety of fresh, processed, and frozen fruits and vegetables; low-fat dairy products; healthy staples and frozen meals; healthy food incentive programs; items sold in check-out areas; portion/cup sizes; and pricing. Each audit item was scored using a five-point semantic-differential scale (1 = provides little or no support for healthful foods to 5 = provides high support for healthful foods). Convergent validity was examined by comparing the SHELF audit to Ghirardelli et al. and Laska et al. store audits. Statistical analysis included: Factor analysis, ANOVA, and Spearman correlations. *Results* SHELF included three factors: a Fruits/Vegetables scale (eight items, α = 0.79; total potential points = 34); a Healthy Foods scale (four items, α = 0.72; total potential points = 16); and a Supports scale (four items, α = 0.685; total potential points = 16). Only 6% of the 124 convenience stores assessed scored in the most healthful range (46–66). The assessed drug stores (*n* = 15) scored higher than convenience stores (*n* = 81) on the Healthy Foods and Supports scales but not the Fruits/Vegetables scale. The SHELF sub-scores were highly correlated with other audit tools indicating convergent validity. *Conclusion* The SHELF convenience store audit is a valid, reliable tool for assessing the degree to which convenience stores support healthfulness regarding Fruits/Vegetables, Healthy Foods, and Supports for choosing healthy.

## 1. Introduction

Over one-third of Americans are obese, and it is well recognized that contributing factors include unhealthy dietary patterns and food choices [1]. The United States Surgeon General [2] urges researchers to evaluate environments with regards to healthfulness of food and nutrition because of the association between the environment, access to healthy food, and consumer food choices [3,4,5,6]. The American population relies on more than supermarkets for their grocery needs. One can buy food at a variety of convenience type stores: gas stations, bodegas, mini-marts, and dollar and drug stores.

Convenience stores are frequented often by a variety of populations [3,7,8]. Over 70% of a New York City (NYC) sample shopped at bodegas five or more times a week, while, at least a third did all of their food shopping at the bodega [3]. Only 40% of the NYC bodegas had any fresh produce [3]. Millennials are shopping more on demand as their use of convenience stores has increased from 7.7% in 2006 to 11.1% in 2014 [9]. Close to 50% of a sample of college students living off-campus purchased three or more food/beverage items from convenience stores on a weekly basis, negatively affecting their fat and sugar intake [7]. Nearly half of an adolescent population reported visiting convenience stores at least weekly [8], and the healthfulness of stores surrounding schools has typically been sub-par [10,11,12]. The food environment affects the population’s health [3,4,5,6], so it is important to accurately document the consumer food environment of convenience stores to enhance efforts to improve healthy food access.

Tools that assess products sold in stores help to describe the consumer food environment [13,14]. These tools measure availability, variety, and price of healthy and less healthy food options as well as store promotions and store environment [15,16,17]. Most tools that assess food store environments use both observational (i.e., freshness of produce) and quantitative measures (i.e., price).

Currently, few comprehensive observations of the consumer food environment of convenience stores have been conducted [15,18,19]. Assessing the consumer food environment may provide valuable findings that can be used to support policy recommendations and interventions that promote healthy food access. Most of the available tools for assessing convenience store consumer food environments present a high time-burden for researchers [18,20,21,22], or are too brief to allow for a comprehensive understanding of the environment [23,24]. Some tools exclude convenience stores, such as drug stores [21]. Few published tools assess the healthfulness of a convenience food store in a practical, convenient, and informative manner. This paper describes the development, reliability and validity of a novel, easy-to-use tool—the Convenience Store Supportive Healthy Environment for Life-promoting Food (SHELF) Audit This tool can be used to assess varied convenience type stores in community and school environments. Data are entered online and the user is provided with results compared and benchmarked to a wider sample of data.

## 2. Methods and Results for Instrument Development

### 2.1. Overview

This study had two components. Study one developed an inventory of items for the audit; conducted expert, cognitive, and pilot tests; and conducted factor analysis which led to a revised survey. For study two, the developed tool (i.e., SHELF) was validated via comparison to two audit tools [25,26]. Data were collected in 2017 on 124 stores on and around geographically diverse college campuses in the Northeast, Midwest, and Southern United States and analyzed in 2018.

### 2.2. Methods: Instrument Development

#### 2.2.1. Development of Inventory Items for the Audit

This audit was designed to rate the small convenience-type food store environments. It can be used to simply evaluate one store or to understand the entire food store environment by evaluating a sampling of stores in a community or defined geographic area.

When consumers make food-related decisions, nutrition information is only one piece of the data considered and may or may not be used [27]. Therefore, this audit is based upon the literature [5,23,25,26,27,28,29,30,31,32,33,34,35,36,37,38] regarding key food store environmental variables (extensiveness of fruits and vegetables, availability of healthier foods, health promotion initiatives, product pricing and placement) known to influence consumer behavior. Thus, the audit was designed to evaluate the array of healthier foods sold in a store and the availability of other supports for making healthier food purchasing decisions (see Table 1). Audited items included: variety of fresh (and quality), processed, and frozen fruits and vegetables; low-fat dairy products; healthy staples and frozen meals; healthy food incentive programs; items sold in check-out areas; portion/cup sizes; and pricing. Each audit item was scored using a five-point semantic-differential scale (1 = provides little or no support for healthful foods, 5 = provides high support for healthfulness).

#### 2.2.2. Store Venues

This study considered the following types of convenience food stores.

- **Convenience store/mini-mart:** These stores sell food items and snacks, limited grocery items, have ≤2 cash registers, and may sell gas [25].
- **Drug store:** These stores sell primarily self-care items, pharmacy items, and limited food and grocery choices.
- **Dollar/discount store:** These stores sell a wide variety of household, personal care, and food items at very low prices. They are smaller than high volume discount “box” stores.
- **Bodega/corner store:** These stores sell mostly food products, including milk, but do not specialize in any one item and have ≤2 cash registers [38].
- **Food cart:** These are mobile food vendors that may or may not be in the same location daily. They sell canned/bottled drinks, packaged snack foods, and similar foods. Food carts that serve only prepared food (e.g., sandwiches, hot pretzels, tacos), were not assessed.

#### 2.2.3. Expert, Cognitive, and Pilot Testing

Audit items were reviewed for content validity by five public health experts and cognitively tested for interpretation and clarity with seven research assistants. Results were used to clarify wording of audit items and response items. The audit was pilot-tested twice. The first pilot test was performed in the summer 2014 in north-central New York state to determine suitability of audit for varied store types and the appropriateness of the range of semantic differential responses.

#### 2.2.4. Pilot Testing Training and Interrater Reliability (IRR)

Student researchers at the participating universities completed the online video training designed to familiarize them with each audit question, the protocol for data collection and potential differences between store types. The student researchers practiced using the audit with fidelity and IRR. Each participating campus team had student researchers complete IRR on two convenience stores with this tool. To pass IRR, interclass correlations >0.80 were expected for each team prior to data collection.

#### 2.2.5. Pilot Test Results

Each team (≥3 student researchers) effectively passed IRR, with appropriate inter-class correlations ICC results (0.821 to 1.0). In Fall 2014, the audit was pilot tested at 12 campuses in the mid-west, northeast, and southeast (*n* = 165) (data not shown). Pilot test findings improved training, IRR protocol, clarified store access, and the audit question regarding beverages.

#### 2.2.6. Audit Administration Procedures

This audit was tested at convenience stores on and near college campuses (*n* = 23). Campuses participating in the Get FRUVED grant (*n* = 70), decided if they wanted to collect SHELF data. Get FRUVED is a social marketing and environmental change intervention to promote health on college campuses. Teams of student researchers and faculty at participating colleges decided which food store establishments were most frequented by their campus population. The team decided the representative sample of convenience stores within a 1.5-mile radius of campus, given this is where students live. The team might have included a store beyond the 1.5-mile radius for evaluation because it was frequently used by the campus population. The Institutional Review Board at Syracuse University determined this research to be exempt because these store audits were environmental, not human research. All data were entered into an online Qualtrics survey link.

#### 2.2.7. Training and IRR

Student researchers at the participating universities completed the online video training designed to inform the student researchers of each audit question, the protocol for data collection, and potential differences between store types. The student researchers practiced using the audit with fidelity and established IRR by taking an online quiz. Each participating student researcher had to pass the quiz with a score of 80% prior to data collection.

### 2.3. Data Analysis

A Bayesian multilevel factor analysis (CFA) was conducted in Mplus software 8.1 [39] to assess the factor structures of the SHELF audit across campuses. Multilevel modeling was applied to address the hierarchical feature of data that stores were nested within college campus environments. The Bayesian approach eliminates small sample and unequal group size problems and provides accurate estimates in multilevel structural equation modeling [40]. Missing data were addressed by using Full Information Maximum Likelihood estimates. Model accuracy was investigated with posterior distribution by using the Markov Chain Monte Carlo (MCMC) method. Multiple chains of the Gibbs sampler were requested and comprised 50,000 iterations, where the first 25,000 were discarded as burn-in, and the remaining 25,000 will be used to estimate the posterior distribution. Chain convergence was monitored by using potential scale reduction factor (PSR), in which PSR less than 1.01 indicates model convergence, trace and density, autocorrelation, and posterior predictive checking scatter plots were recorded (not reported but available upon request). Non-informative priors were used and model fit was assessed by using posterior predictive P-value (PPP), and a 95% confidence interval for the difference between the observed and replicated chi-square value. A Chi-square value around zero with 95% confidence intervals (CIs) that encompasses zero and a PPP value around 0.50 indicate good model fit, whereas larger chi-square values with 95% CIs that do not encompass zero and a PPP value less than .05 indicate poor model fit [41]. Posterior means, posterior standard deviations, and 95% CIs were computed and are reported.

To determine the healthfulness of the stores latent profile analysis (LPA) was applied by using three subscales (fruit/vegetable, healthy foods, supports) of the SHELF audit. The model building started from two profiles, and the following models were tested iteratively. Robust maximum likelihood method was used and several criteria were used to guide the best solution: Bayesian Information Criteria (BIC), Lo–Mendell–Rubin Likelihood Ratio Test (LRT), Bootstrapped Likelihood Ratio Test (BLRT), entropy, sample-size adjusted BIC (SSABIC), and the uniqueness and interpretability of latent profiles [42]. Lower BIC and SSABIC values indicated better model fit. Entropy assessed the classification accuracy and higher entropy values indicated better model fit. The LRT and BLRT with a significant p-value suggested the model fits better than the model with one less class.

### 2.4. Results for Instrument Development

#### 2.4.1. Multilevel Factor Analysis

A total of 124 stores were assessed with SHELF. Most stores were convenience (*n* = 81, 65.3%) followed by corner stores (*n* = 17, 13.7%) drug stores (*n* = 15, 12.1%), and dollar/discount stores (*n* = 11, 8.9%).

Three factors were specified in the factor analysis model by using non-informative priors for factor loadings (N~0, 0.01). The Fruits/Vegetables construct included eight items (fresh fruit and vegetable availability and quality; processed and frozen fruit and vegetable availability), the Healthy Foods construct included four items (low-fat dairy, healthy staple foods, healthier pre-packaged frozen meals, and healthy products adjacent to checkout), and the Supports construct included four items (pricing of processed fruit and vegetables and prepackaged frozen meals, and healthier food incentive programs). The model showed a good posterior predictive model fit with the 95% CIs for the difference between the observed and replicated chi-square values covered zero [−36.046, 68.627] and the posterior predictive *p*-value was 0.279. Factor loadings range (see Table 2) from 0.23 to 0.95 on the Fruits/Vegetables scale (α = 0.793; total potential points = 34), 0.25 to 0.74 on the Healthy Foods scale (α = 0.721; total potential points = 16), and 0.44 to 0.90 on the Supports scale (α = 0.685; total potential points = 16). Low factor loadings were maintained given they were each significant within the model.

#### 2.4.2. Results Describing the Healthfulness of Stores

Table 3 presents the model fit indices for two- to four-class latent profile analysis (LPA) solutions of the healthfulness of stores. The LRT indicated two-class solution was a better while the BLRT was significant for the two-, three-, and four-class solutions. The BIC and SSABIC were smaller for the four-class solution. However, the three- and four-class solutions had a very similar structure SHELF sub-scores. Results of the three-profile model are presented in Table 3. The three-profile model was identified as the best solution based on fit indices and meaningful interpretation of profiles. The first profile (least healthy) consisted of 45.1%, the second profile (moderately healthy) consisted of 48.3%, and the third profile (most healthy) consisted of 6.4% of the sample (means and ranges available in Table 4).

Table 4 conveys that most stores were located in the southern region of the US (51.2%) followed by the northeast region (36.5%), only 4.8% of the stores evaluated were in the western region of the US. Although there were no differences on the Fruits/Vegetables factor by store type, convenience stores scored significantly lower than drug stores on the other two factors (Healthy Foods and Supports). Bodegas also scored significantly lower than drug stores on the Healthy Foods factor as shown in Table 5.

## 3. Methods and Results for Validation Study of SHELF

### 3.1. Methods for Validation Study

Most store audits rely only on expert validation and reliability results in the development process. To adequately test SHELF, convergent validity was assessed through comparison with similar tools. A sample of stores (*n* = 92) was evaluated from eight universities for this validation study in 2016.

#### 3.1.1. Comparison Tools

The Laska et al. [26] tool was selected because it contained a varied checklist of 28 food and beverages. The items on this audit included: fresh fruits and vegetables (including pre-packaged salads), processed fruits and vegetables (including frozen or canned (light-syrup or juice only, no-added sugar apple sauce); healthy beverages (water, 100% juice, low-fat dairy (1% or skim milk), low- or reduced fat cheese/yogurt (<10% Daily Value (DV) fat)); healthy snacks (nuts, low-fat crackers, popcorn, trail-mix/dried fruit (< 10% DV fat), low-sugar/fat granola bars (<10% DV sugar/fat), pretzels, baked chips and graham or animal crackers); and other healthy staples (peanut butter, high-fiber bread/cereal (>10% DV fiber), beans/lentils, brown rice, low-sugar cereal or pudding (<10% DV sugar), fruit-flavored gelatin, and pre-made sandwiches). The checklist is yes/no for availability in general and for single serving package size. The supports for healthy choices included questions about ads/promotions for food/beverage, tobacco, and alcohol in the checkout area, hanging from ceiling, or outdoors; and items available at the checkout, such as unhealthy (gum/candy, soda, chips, other) versus healthful (granola bars, bagged nuts/seeds, fresh fruit, water, other). To enable comparison of Laska et al. [26] checklist results to the SHELF results, the simple checklist was converted into three sub-scores: (1) Fruits/Vegetables sub-score (additive): “Yes” for fresh veggies, fresh fruit, prepackaged salad, 100% juice, frozen or canned veggies, canned fruit, no-added sugar applesauce), (2) Healthy Foods sub-score (additive): “yes” checked for non-fruit and vegetable foods, “no” checked for neutral foods (diet soda, sandwiches, pudding, penny candy) or for soda, chips. (3) Supports sub-score (additive): “Yes” healthy signage, accepts Women Infant and Children (WIC) benefits, healthy foods at check out and “No” for unhealthy foods at check out. Maximum sub-scores = Fruits/Vegetables sub-score (0–14), Healthy Foods sub-score (0–22), and Supports sub-score (0–12).

The California Department of Public Health Communities of Excellence in Nutrition, Physical Activity, and Obesity Prevention (CX3) Food Availability and Marketing Survey was also selected to validate SHELF.CX^3^ checklist [25] was selected for validation because it contained a more extensive evaluation of the condition and marketing of products, health promotion programs, availability of local produce, quality of fruits/vegetables, and the availability of 33 food and beverage items. Specifically the audit includes fresh fruits and vegetables, processed fruits and vegetables (frozen or canned (no-added fat/sugar/sweetener)); other health foods (low-fat dairy (2%, 1% or skim), mozzarella part-skim, ground beef/turkey (85% lean), whole chicken, whole wheat bread, brown rice, high-fiber cereal (>3 gm, ≤12 gm sugar/serving), oatmeal (plain), tortillas (soft corn or whole wheat), soy beverages (plain no added sugar and flavored), tofu plain, beans (dried and canned (no added fat sugar, etc.)), tuna (light in water), salmon (canned in water), sardines (canned in water), and jarred baby food (single fruit, vegetable, meat)). Available at the checkout: unhealthy (candy, soda, chips, other) versus healthful (granola bars (≥2 gm fiber, ≤1 gm sat-fat, ≤14 gm sugar/serving), bagged nuts/seeds, fresh fruit, water, other). To enable comparison of the CX^3^ checklist results to the SHELF results, the simple checklist was converted into three sub-scores: 1) Fruits/Vegetables sub-score (additive): four scale questions assessing variety and quality of fresh fruits and vegetables “Yes” for fresh fruit/vegetables (7 items), healthy processed fruits/veggies (4 items), 2) Healthy Foods sub-score (additive): “Yes” checked for non-fruit and vegetable foods, excluding baby food questions, 3) Supports sub-score (additive): “Yes” healthy signage, accept WIC/Supplemental Nutrition Assistance Program (SNAP) benefits, healthy foods at check out and “No” for unhealthy foods at check out, and health promotion programs available. Maximum sub-scores = Fruits/Vegetables sub-score (0–17), Healthy Foods sub-score (0–19), and Supports sub-score (0–22).

#### 3.1.2. Procedure

Training and IRR: In addition to the standard SHELF training and IRR protocol as outlined above, research assistants completed training, practiced, and performed IRR with Laska et al. [26] and CX^3^′s checklist tools before collecting data. Each participating community had the student researchers complete training and establish IRR by using each tool at two different stores. Each team of students (≥3 research assistants) had to achieve IRR greater than 0.80 for each tool to prior to commencing data collection.

For validation purposes, each team assessed a variety of convenience store types using all three tools (SHELF, Laska et al. [26], and CX^3^). To reduce potential bias, Laska et al. [26] and CX^3^ were collected first for one half of the audits, and for the other half, SHELF was collected first. All surveys were entered into Qualtrics, with one for SHELF and one for Laska et al. [26] and CX^3^ combined.

### 3.2. Data Analysis

The SPSS statistical software package version 24.0 was used for data analysis. Interclass correlations (ICCs) were calculated to determine student researcher IRR; Spearman correlations were used to compare Laska et al. [26] and CX^3^ Fruits/Vegetables, Healthy Foods, and Supports sub-scores with that of SHELF factors. Analysis of variance with Tukey’s Honestly Significant Difference (HSD) post hoc statistics was conducted to determine differences between communities and venues. Significance level was set at *p* < 0.05.

### 3.3. Results of Validation Study

Validation study results are in Table 6 and Table 7. Each team effectively passed IRR, with appropriate ICC results (0.821 to 0.997). A total of 92 stores were assessed with SHELF, Laska et al. [26], and CX^3^ checklist tools for validation. Most stores were convenience (*n* = 63, 70%) followed by corner stores (*n* = 14, 15.2%) drug stores (*n* = 11, 12%), and dollar/discount stores (*n* = 2, 2.2%). There were significant correlations (*p* ≤ 0.001) between SHELF and Laska et al. [26] for both Fruits/Vegetables (*r* = 0.717) and Healthy Foods (*r* = 0.748), but not for the Supports sub-score (*r* = 0.125, *p* = 0.51). As for SHELF and CX^3^, all sub-scores were significantly correlated (*p* ≤ 0.001) for Fruits/Vegetables (*r* = 0.876), Healthy Foods (*r* = 0.767), and Supports (*r* = 0.383).

## 4. Discussion

The SHELF convenience store audit was developed using a multiphase systematic development process. The audit inventory items were judged as understandable and logical based upon cognitive and pilot-tests. High IRR results indicated a consistency between evaluators. Factor analysis of the convenience store results determined a three-factor structure to provide effective sub-scores for Fruit/Vegetables, Healthy foods, and Supports for choosing healthy items. The SHELF distinguished the healthfulness of the convenience store environment with only 6.4% scoring in the most healthful category. The SHELF audit was determined to be valid based on expert convergent comparisons.

Given the high prevalence and accessibility of convenience stores in urban [3,12,20,43] and rural environments [23,28,44], valid and reliable, yet practical tools for assessing and comparing convenience stores are necessary. The SHELF can fill this role. Using SHELF, we found almost 95% of the stores in this study scored low to moderately healthy on the Fruits/Vegetables sub-scale, implying low fresh, frozen and processed fruits and vegetables. Similarly, a more complex convenience store audit in Minnesota [43] found 62% carried one or more varieties of fresh fruit, and only one-third stocked one or more varieties of fresh vegetables. Significantly fewer stores stocked at least three varieties or more [43]. Although 50% of the convenience stores in another study [12] carried some fresh fruit and vegetables, significantly lower percentages were available as single-serve items. Similar to our study, their convenience stores [12] had low percentages of healthy beverages and snacks available (low-fat yogurt, etc.) and supports for choosing healthfully.

In this study, there were no differences on the Fruit/Vegetable sub-score by store type. However, drug stores outperformed convenience stores and bodegas on the Healthy Foods, and drug stores scored higher than convenience stores on Supports. In contrast, another audit [45] found 63% of convenience stores (which includes small grocery stores) commonly sold fresh vegetables versus 23% of drug stores, 8% of food-gas marts, and zero dollar stores. Food-gas marts were the most likely to sell fresh fruit (71%), followed by convenience stores (61%). Few drug stores stocked fresh fruits or vegetables (23%), and no dollar stores stocked fresh produce. In general, drug stores and dollar stores stocked a narrower range of healthy options, but more consistently had canned fruits and vegetables and whole grain-rich cereal, compared with other types of stores.

The SHELF audit was determined to be valid based on expert review and comparison to Laska et al. [26] and CX^3^ [25] convenience store tools. It was highly consistent when comparing the Fruits/Vegetables and Healthy Foods scales. Only CX^3^, but not Laska et al. [26], positively and significantly correlated with SHELF for Supports for choosing healthfully. This latter finding may be because the Laska et al. [26] tool was less specific than the CX^3^ regarding the healthfulness of the environmental supports. Glanz [15] reported most existing food store audits have tested reliability, but very few had assessed validity. A strength of this study is the convergent validation with other tools.

The SHELF audit is a novel tool for community and researchers to assess the consumer food environment using a streamlined, reliable, valid tool thereby offering an alternative [11,24,25,26] particularly for the more time-intensive tools [18,20,21,30]. Although the SHELF audit and an existing convenience store audit [18] can be used to assess and distinguish between a variety of convenience store types (drug store, dollar store, gas station), SHELF is more streamlined at 22 semantic differential questions versus a 12-page survey. The SHELF training and practice require approximately 2–3 h and an audit can be completed in 25–30 mins. The SHELF audit is part of the Healthy Campus Environmental Audit (HCEA), a series audit tools to evaluate restaurants [46], vending machines, recreation services, walkability/bike-ability, and policies.

The SHELF is available on the internet with training and data entry links (contact the primary author for information). The easy-to-use training and online data entry are user friendly. The data are analyzed by the primary institution and feedback is provided to the user including comparison and benchmark information. A significant limitation of this study is that the audit has been used and tested by college-educated populations on and near college campuses, thus it needs to be validated for use by other data collectors in varied communities. Finally, the SHELF audit should be qualitatively validated by comparing audit results with consumer perceptions as well as consumer purchasing behavior.

## 5. Conclusions

This easy to use SHELF Convenience Store Audit effectively assessed the Fruits/Vegetables, Healthy foods, and environmental Supports for choosing healthfully and allowed for comparison between a variety of convenience store types. It could be used to assess convenience store environments near schools and other communities regarding baseline status, tracking over time, or making comparisons between communities. Nutrition educators can use the SHELF Convenience Store Audit with groups and can use the results to educate them on how to make wise decisions or to advocate for healthier choices given the realities of the convenience store healthfulness. The SHELF Convenience Store Audit provides valuable information to advocate for policy changes to help consumers who make quick choices to find it easier to make healthful choices. A few examples of policy recommendations might include: providing more fruits and vegetables at a reasonable price, banning unhealthy products at the check-out counter, and a sustainability/healthy food tax incentive [31].

## Figures and Tables

**Table 1:** SHELF Audit Question ^1^.

Categories	Questions	Instructions	Scoring
Fruit and Vegetables (*n* = 10)	How many varieties of fresh fruit are available? [23,25,26,28,30,33]	Examples: Whole, fresh fruit, Cut and packaged fresh fruit Instructions: Count only fruit types (i.e., “apple”) and not individual variations of each fruit (i.e., Granny Smith vs. Red Delicious).	None, 1–3, 4–6, 7–9, ≥10
	How many varieties of fresh vegetables are available? [23,25,26,28,30,33]	Examples: Whole fresh vegetables, Cut and packaged fresh vegetables *Prepared vegetable-based salads Instructions: *Count only the vegetable type (i.e., “carrots”) and not individual variations of each vegetable (“baby carrots” plus “whole carrots”).	
	Which statement best describes the quality of the fresh fruit? [23]	Poor quality: bruised, overripe Good quality: fresh, not overripe, few blemishes Answers: All or must fruit/vegetable are poor quality, bruised or over ripe (wilted); slightly more poor quality fruit/vegetables (bruised overripe) than good quality; mixed equal proportion of poor and good quality; slightly more good quality fruit/vegetables than poor quality; All or most of fruit is good quality, fresh, not overripe, low blemishes.	Poor quality to high quality, 1 to 5
	Which statement best describes the quality of the fresh vegetables? [23]		
	How many varieties of processed fruits are available? [23,25,26,30,33]	Examples: Canned or cup of fruit: single or mixed, Dried fruit, Applesauce Instructions: Count only types of fruit for each category. Ex: If multiple container sizes and brands are available for canned pineapple, only count pineapple once. Count “regular” and “light” options of the same fruit separately. If multiple flavors of applesauce are present, count applesauce only once.	None, 1–5, 6–10, 11–15, ≥16
	How many varieties of processed vegetables are available? [23,25,26,30,33]	Examples: Canned or jar vegetables: single vegetable or mixed Instructions: Count only types of vegetable as one variety. If there are multiple size cans or brands, count the vegetable only once. Count “regular” and “low sodium” versions of the same vegetable as separate choices. Do not include pickles or olives.	None, 1–3, 4–6, 7–9, ≥10
	Which statement best describes the pricing of processed fruits? [23,29]	Definition: “Light” variety is packed in water, fruit juice, or light syrup Instructions: Look at identically-sized cans of the same fruit, one “regular” and one “light” Compare the price of the two varieties. If there are no “regular” and “light” varieties of a singular fruit type available, two different fruits in the same size container can be compared.	No healthy options available, all healthy comparisons cost more, most of healthy comparison cost more, Healthy and Unhealthy are equally priced, more of healthy cost less, all healthy comparisons cost less.
	Which statement best describes the pricing of processed vegetables? [23,29]	Instructions: Look at identically sized cans of the same vegetable, one “regular” and one “low sodium.” Compare the price of the two varieties. If there are no “regular” and “low sodium” varieties of a singular vegetable type available, two different vegetables in the same size container can be compared	
	How many varieties of frozen fruits are available? [23,25,26,30,33]	Examples: Single or mixed vegetable types frozen in box or bag Don’t count frozen entrees/meals that contain vegetables.	None, 1–3, 4–6, 7–9, ≥10
	How many varieties of frozen vegetables are available? [23,25,26,30,33]		
Low fat Dairy	How many varieties of low-fat dairy products or dairy substitutes are available? [23,25,26,28,33]	Examples: Low-fat or nonfat cow’s milk, soy milk, almond milk, or lactose-free milk (skim or 1%; plain or flavored), Low-fat or nonfat yogurt (cow’s milk or soy), Low-fat cheese (single serving or brick, cow’s milk or soy) or cottage cheese (< 4% milkfat) Do not include: Eggs, Cream (i.e., fat-free half and half), Muscle Milk or other high-calorie milk blends, butter, ice cream or other dairy-based desserts Instructions: Count all sizes and brands of a dairy category as one variety (i.e., if one-gallon sizes and half-gallon sizes of 1% milk are available, count only as one choice.) However, different “flavors” count separately (i.e., chocolate 1% milk and plain 1% milk would count as two choices.) Count all yogurt flavors as one choice. Look on the shelves (outside the coolers) for dairy alternatives such as almond milk.	None, 1–3, 4–6, 7–9, ≥10
Staples	How many varieties of the following healthy staple foods are available? [23,25,26,28,33]	Examples (per serving): High fiber bread* products (≥10% DV fiber): bread*, bagels, English muffins, tortillas, etc. Cereal that is high in fiber (≥10% DV) **AND** low in sugar (<10g unless containing dried fruit) Examples: Whole wheat toasted oat cereal (i.e., original Cheerios), Fiber One, Quick cooking oatmeal—plain. Always include 100% whole wheat bread, even if it doesn’t meet the fiber DV requirement Instructions: Count each individual flavor or food type as one choice. (i.e., if more than two brands of 100% whole wheat bread are present, count only once). If more than one brand of a type of cereal are present, count the cereal type only once. (i.e., generic brand oatmeal and Quaker oats count as one choice.)	None, 1–5, 6–10, 11–15, ≥16
Frozen Meals (*n* = 2)	How many varieties of healthier prepackaged frozen entrees/ meals are available? [7,23,25,26]	Examples: Frozen pizza (single cheese or veggie), Frozen burritos, Frozen single or multi-portion meals Brands or types to look for: Lean Cuisine, Amy’s, Healthy Choice, Smart Ones, Kashi, Lean Pockets, Amy’s, Glutenfreeda, Evol, Cedarlane brand frozen burritos Instructions: Count each individual entrée type as one choice, even within the same brand. Healthier meals should meet the following criteria: ≤500 calories per meal **AND** ≤ 35% DV fat per meal/serving. **Plus**, they must meet 5 of the following: Saturated fat: ≤10% DV; Trans-fat: 0; Fiber, Vitamin A, Vitamin C, Calcium, Iron: ≥10% DV; Sodium ≤600 mg; Sugar: ≤12.5 g; Protein: ≥10g.	None, 1–3, 4–6, 7–9, ≥10
	Which statement best describes the pricing of the prepackaged frozen entrees/meals? [23,29]	Instructions: Find “regular” versions and “healthier” versions of the same entrée. They should be generally the same size. (i.e., 12” pepperoni pizza vs. 12” cheese pizza)	No healthy options available, all healthy comparisons cost more, most of healthy comparison cost more, Healthy and Unhealthy are equally priced, more of healthy cost less, all healthy comparisons cost less.
Largest Beverage size	Which is the best description of the largest cup size available for self-service or fountain drinks? [31]	Answers range from >3 cup sizes available larger than 16 oz. OR are all cup sizes available are > 16 oz. down to largest size is 16 oz.	All cup sizes available are > 16 oz., 3 cup sizes > 16 oz., 2 cup sizes are > 16 oz., 1 size is > 16 oz., and Largest size is 16 oz.
Check out items (*n* = 2)	How many healthy products are adjacent to the checkout counter? [23,25,26]	“Adjacent” means: On racks directly beneath or connected to checkout counter; On racks in the checkout aisle; In a cooler on an endcap connected to a checkout aisle Examples: Bottled water; low-fat dairy, Fresh fruit or vegetables, Dried fruit, Nuts/seeds, Granola/cereal bars containing < 10g sugar & <10% DV for fat per serving^2^ Instructions: Count only categories here. For example, if more than one brand of bottled water is present, count only once.	None, 1–3, 4–6, 7–9, ≥ 10
	How many unhealthy products are adjacent to the checkout counter? [7,23,25,26]	Examples: Candy, Sugar-sweetened drinks, Energy drinks, Chips/other high-fat and sodium snack foods, Cookies/other packaged baked goods, Beef jerky	≥ 10, 7–9, 4–6, 1–3, None
Healthy Choice Pro-grams	What programs are in use to advertise healthy choices? [34,35,36]	Examples: Guiding Stars program, “Healthy Option” labels on shelving, MyPlate illustrations. Do not count Gluten-free labels, vegan or vegetarian labeling	None,1,2,3, ≥ 4
Access (*n* = 3)	Approximately how far is this store from the geographic center of campus? [5]	Answers include: accessible by car, public transportation, bike, walk (2/3–1 mile) walk (<2/3 mile)	Select all that apply-additive score [1,5]
	What is the average number of hours this store is open on Tuesdays? …. on Sundays? [32,37]		<3, 4–6, 7–9, 10–12,>12 h

^1^ Source of questions noted for each question. SHELF = the Convenience Store Supportive Healthy Environment for Life-promoting Food.

**Table 2:** Factor Analysis Results ^a^.

SHELF Factors	Estimate	Posterior S.D.	One-tailed *p*-value	95% CI
Fruit and Vegetable ^b^				
Fresh fruit availability	0.69	0.07	<0.001	(0.55, 0.81)
Fresh fruit quality	0.38	0.16	0.019	(0.02, 0.63)
Processed fruit availability	0.79	0.06	<0.001	(0.64, 0.87)
Frozen fruit availability	0.92	0.04	<0.001	(0.81, 0.97)
Fresh vegetable availability	0.69	0.09	<0.001	(0.47, 0.83)
Quality of fresh vegetable	0.23	0.09	0.010	(0.04, 0.41)
Processed vegetable availability	0.62	0.09	<0.001	(0.42, 0.76)
Frozen vegetable availability	0.95	0.03	<0.001	(0.87, 0.98)
Healthy Foods ^c^				
Low fat dairy	0.70	0.09	<0.001	(0.50, 0.81)
Healthy stable foods	0.74	0.08	<0.001	(0.55, 0.87)
Healthy prepackaged frozen meals	0.71	0.08	<0.001	(0.53, 0.85)
Healthy products adjacent to checkout	0.25	0.12	0.019	(0.01, 0.47)
Supports ^d^				
Pricing of processed fruits	0.64	0.08	<0.001	(0.47, 0.78)
Pricing of processed vegetables	0.90	0.06	<0.001	(0.73, 0.97)
Pricing of prepackaged frozen meals	0.46	0.13	<0.001	(0.18, 0.69)
Programs	0.44	0.13	0.002	(0.16, 0.68)

^a^ Factor Analysis: 95% CIs for the chi-square values: [−36.046, 68.627], and posterior predictive P- (PPP)-value = 0.279. ^b^ Fruit and Vegetable sub-scale 8 items (α = 0.793) (total possible points = 34). ^c^ Healthy Foods sub-scale 4 items (α = 0.721) (total possible points = 16). ^d^ Support sub-scale 4 items (α = 0.685; total potential points = 16).

**Table 3:** Fit statistics for latent profiles.

Class	BIC	SSABIC	LRT (*p*)	BLRT (*p*)	Entropy
2-class	2153.260	2121.640	114.023 (<0.001)	114.023 (<0.001)	0.858
3-class	2129.259	2084.990	41.148 (0.09)	43.282 (<0.001)	0.870
4-class	2118.711	2061.794	28.358 (0.12)	29.829 (<0.001)	0.841

BIC: Bayesian Information Criteria; SSABIC: sample-size adjusted BIC; LRT: Lo–Mendell–Rubin Likelihood Ratio Test; BLRT: Bootstrapped Likelihood Ratio Test.

**Table 4:** SHELF audit administration demographics.

Distribution	All Stores	Least Healthy	Moderately Healthy	Most Healthy
	*n* = 123	*n* = 56	*n* = 59	*n* = 8
Location				
Northeast	45	13.70% (17)	18.54% (23)	4.03% (5)
Midwest	10	4.83% (6)	3.22% (4)	0
South	63	23.38% (29)	25% (31)	2.41% (3)
West	6	3.22% (4)	1.61% (2)	0
Sub-Scores				
Fruits/Vegetables		7.07 ± 4.68 (0, 20)	13.2 ± 5.35 (4, 28)	29.38 ± 4.10 (23, 33)
Healthy Foods		4.86 ± 2.59 (0, 10)	10.18 ± 2.23 (6, 15)	14.88 ± 1.46 (13, 17)
Supports		2.86 ± 1.89 (0, 7)	8.47 ± 2.55 (3, 13)	11.38 ± 1.77 (10, 14)

SHELF = the Convenience Store Supportive Healthy Environment for Life-promoting Food.

**Table 5:** Differences in SHELF sub-scores by store type.

Sub-Scores	Convenience	Drug Store	Dollar/Discount	Bodega/Corner	Total
	*n* = 81	*n* = 15	*n* = 11	*n* = 17	*n* = 124
	Mean ± SD	Mean ± SD	Mean ± SD	Mean ± SD	Mean ± SD
Fruit/Vegetables	11.90 ± 8.52	10.87 ± 4.39	9.27 ± 5.22	11.41 ± 5.12	11.48 ± 7.46
Healthy Foods ^1^	7.57 ± 3.93 ^a^	11.93 ± 1.87 ^b^	8.18 ± 4.56	7.06 ± 2.95 ^a^	8.08 ± 3.92
Supports ^2^	5.31 ± 3.75 ^a^	9.33 ± 2.16 ^b^	7.0 ± 3.95	6.59 ± 3.37	6.12 ± 3.77

^1^ F-test (3,123) = 6.419, *p* ≤ 0.0001; ^2^ F (3,123) = 5.768, *p* ≤ 0.001; ^a,b^ Within a factor, different superscripts are significantly different between store types at *p* < 0.05.

**Table 6:** Validation study descriptive statistics (*n* = 92).

Audit Variables	Mean ± SD	Range
SHELF Fruit/Vegetables	7.23 ± 6.88	(0, 28)
SHELF Healthy Foods	4.66 ± 3.26	(0, 13)
SHELF Supports	3.67 ± 2.67	(0, 12)
Laska et al. Fruit/Vegetables	5.73 ± 2.69	(0, 12)
Laska et al. Healthy Foods	13.39 ± 4.23	(3, 20)
Laska et al. Supports	3.99 ± 1.34	(1, 7)
CX^3^ Fruit/Vegetables	9.5 ± 6.78	(0, 25)
CX^3^ Healthy Foods	7.0 ± 3.93	(0, 17)
CX^3^ Supports	8.08 ± 2.23	(2, 14)

SHELF = the Convenience Store Supportive Healthy Environment for Life-promoting Food. CX^3^ = Communities of Excellence in Nutrition, Physical Activity, and Obesity Prevention Food Availability and Marketing Survey.

**Table 7:** Validation study sub-score correlations.

**Audit Variables**	**SHELF Fruit/Vege**	**Laska et al. Fruit/Vege**	**CX^3^ Fruit/Vege**
SHELF Fruit/Vegetables	1		
Laska et al. Fruit/Vegetables	0.720 **	1	
CX^3^ Fruit/Vegetables	0.898 **	0.753 **	1
	**SHELF Healthy Foods**	**Laska et al. Healthy Foods**	**CX^3^ Healthy Foods**
SHELF Healthy Foods	1		
Laska et al. Healthy Foods	0.731 **	1	
CX^3^ Healthy Foods	0.660 **	0.777 **	1
	**SHELF Supports**	**Laska et al. Supports**	**CX^3^ Supports**
SHELF Supports	1		
Laska et al. Supports	0.125	1	
CX^3^ Supports	0.456 **	0.548 **	1

** *p* ≤ 0.001. SHELF = the Convenience Store Supportive Healthy Environment for Life-promoting Food. CX^3^ = Communities of Excellence in Nutrition, Physical Activity, and Obesity Prevention Food Availability and Marketing Survey.
